# Development of an Inductive Rain Gauge

**DOI:** 10.3390/s22155486

**Published:** 2022-07-22

**Authors:** Christoph Clemens, Annette Jobst, Mario Radschun, Jörg Himmel, Olfa Kanoun, Markus Quirmbach

**Affiliations:** 1 Institute for Measurement Engineering and Sensor Technology, Ruhr West University of Applied Sciences, 45479 Mülheim an der Ruhr, Germany; annette.jobst@hs-ruhrwest.de (A.J.); mario.radschun@hs-ruhrwest.de (M.R.); joerg.himmel@hs-ruhrwest.de (J.H.); 2 Chair for Measurement and Sensor Technology, Chemnitz University of Technology, 09126 Chemnitz, Germany; olfa.kanoun@etit.tu-chemnitz.de; 3 Institute for Civil Engineering, Ruhr West University of Applied Sciences, 45479 Mülheim an der Ruhr, Germany; markus.quirmbach@hs-ruhrwest.de

**Keywords:** smart sensor, internet of things, rain gauge, eddy current, LoRaWAN

## Abstract

Measuring weather data in an urban environment is an important task on the journey towards smart cities. Heavy rain can cause flooding in cities and prevent emergency services from reaching their destination because roads or underpasses are blocked. In order to provide a high-resolution site-specific overview in urban areas during heavy rainfall, a dense measurement network is necessary. To achieve this, a smart low-cost rain gauge is needed. In this paper, the current status of the development of an inductive rain gauge is presented. The sensor is based on the eddy current principle and evaluates the frequency of an electrical resonant circuit. For this purpose, a coil is placed under a metal plate. When raindrops hit the plate, it starts to oscillate, which changes the distance to the coil accordingly and causes changes in the frequency of the resonant circuit. Since the sensor is cost-effective, operates self-sufficiently in terms of energy and transmits data wirelessly via LoRaWAN, it can be used flexibly. This enables dense, area-wide coverage over the urban area of interest. The first experimental investigations show a correlation between the size of the rain droplets and the frequency change. Small droplets cause a shift of about 8 kHz and larger droplets of up to 40 kHz. The results prove that raindrops can be detected and categorized using this measurement principle. These data will be used as a basis for future work on calculating precipitation.

## 1. Introduction

Heavy rain and flash floods are not a new phenomenon. However, extreme weather events, especially in the summer months, are increasing as a result of climate change [1,2]. The sewer networks of urban areas especially can quickly become overloaded and streets and underpasses can become flooded. These rain events typically occur locally and suddenly. Therefore, it is difficult to make an accurate prediction of when and where the rain will occur. Weather warnings concerning heavy rainfall events are issued almost daily during appropriate weather conditions, especially in the summer months. In addition, these warnings apply to a large area, but since heavy rain usually occurs locally, false alarms are more frequent and, thus, there is a risk that they are not heeded. All of these factors make it difficult for emergency services to respond appropriately to warnings. Inaccurate warnings are partly due to the insufficiently dense measurement network for precipitation in cities. With a closer-meshed monitoring network, it would be possible to detect rain events in real time and locally, and then respond accordingly. Furthermore, the direction could be determined in which the precipitation cell is moving. Besides the amount of rain, the size of the raindrops is also important for the evaluation of meteorological events.

The work presented in this paper is embedded in a current research project dealing with AI-based prediction of heavy rainfall and flash floods in urban areas [3]. The aim of the research is to solve the described problem of inaccurate warnings by using AI that predicts the occurrence and course of heavy rain events and marks the flood-prone area. Part of the implementation is to establish a dense sensor network in an urban area. This involves a rain gauge that meets the following requirements.

- Low-cost and low-maintenance due to a high number of units;
- Flexible installation;
- Energy self-sufficient;
- Continuous and wireless data transmission;
- Measurement of raindrop size and amount of rain.

Since there is no sensor on the market that meets these specific requirements, a smart rain gauge based on the eddy current principle and taking IoT aspects into account is to be developed as part of the research project.

This article is structured as follows. First, the state of the art is described. In the materials and methods chapter, the sensor concept and measurement principle are presented. Then, the electrical and mechanical backgrounds of the sensor principle are explained. In the results, the practical implementation of the sensor is presented in terms of the electrical circuit, data transmission and power supply, and the distance between the coil and the plate. Afterwards, a measurement of different droplets is performed to demonstrate the measurement effect. Finally, the work is summarized, and future work and improvements are addressed.

### State of the Art

One known method of determining precipitation and droplet size is based on acoustics. The sensor works similar to a microphone. The impacting rain causes an acoustic impulse, which is converted into an electrical signal by a piezo element. Based on the strength and frequency of the signal, the quantity and size of the drops can be inferred. However, the use of this method is unsuitable during heavy rain events, wind and thunderstorms due to the large amount of background noise [4,5,6].

Another approach is an optical measurement method in which a narrow homogeneous laser band is emitted and picked up by a receiver. If rain falls through the laser band, the intensity at the receiver decreases. Based on the duration and amount of intensity reduction, the droplet frequency and size can be determined. However, other causes such as fog clouds can also lead to a reduction in intensity and adversely affect the measurement principle. In addition, this measurement principle has a high-power requirement due to the laser technology. As a result, sensors of this type can neither operate over a long period of time powered by a rechargeable battery nor are they low-cost and low maintenance [4,7,8].

The Joss–Waldvogel distrometer is based on the induction principle. The rain falls on a cone which starts to oscillate. The oscillation induces a voltage in a coil at the bottom of the cone. Evaluating the voltage, conclusions can be drawn about the rain characteristics. The Joss–Waldvogel distrometer is most similar to the method planned in this project, as it also uses an inductive method. However, the Joss–Waldvogel distrometer evaluates a voltage, which must first be amplified and read in via an A/D converter. This increases the power consumption. Commercially available devices require a power supply of 230 volts AC [4,7,9].

The registered design DE 202020104245 U1 describes a system for analyzing precipitation events based on an energy-autonomous low-cost precipitation sensor, as well as computer program. The sensor uses a capacitive measurement method. The rain falls on one of the capacitor plates which is free-swinging. This varies the distance between the plates and the capacitance of the capacitor changes. Thus, a voltage or frequency change in a resonant circuit can be measured. The capacitive design with two capacitor plates has the disadvantage that environmental influences, such as humidity, have a very large effect on the capacitance. These problems do not occur with an inductive method, as planned in this project [10].

There are also low-cost resistive rain sensors that are often used in cars for automatic wiper control. However, these sensors do not provide information about the drop size or the amount of rain, rather they serve as a status of whether it is raining or not [11,12,13]. In addition, none of the sensors possess a wireless interface for data transmission.

Eddy current-based sensors are common in the industry and find application in different areas such as process monitoring, material testing and production control [14,15,16,17].

The inspiration for the sensor developed here came from a pressure sensor based on the eddy current principle. In this sensor, a coil operated with alternating current is located under a thin conductive metal membrane. The electromagnetic field of the coil generates an eddy current in the membrane. The eddy current generates a counter field. When pressure is applied to the membrane, the membrane bends and the distance to the coil changes. This affects the eddy current and the counter field, which can be measured. This sensor works on the same principle as the planned rain gauge. The difference is that in the rain gauge application, the raindrops trigger a dynamic response from the membrane instead of static behavior due to constant pressure [18,19,20].

## 2. Materials and Methods

### 2.1. Sensor Concept

**Figure 1:**
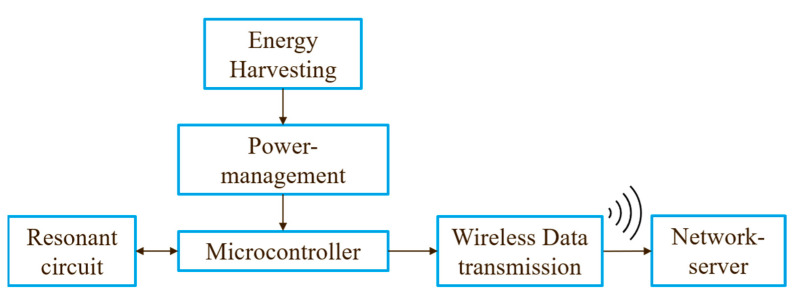
Sensor concept.

Figure 1 shows the concept of the inductive rain gauge currently under development. The measuring principle of the sensor is based on the eddy current principle. For this purpose, an electrical coil is placed under a conductive plate. The coil is operated in a permanent resonant circuit so that it oscillates at the resonant frequency. This generates a periodic magnetic field, which, vice versa, induces an eddy current in the plate. According to Lenz’s law, this induced current counteracts its cause, and a counter field is created, which leads to the attenuation of the magnetic field. Thus, the inductance of the excitation coil is reduced due to the resulting mutual inductance and the resonant frequency is changed.

If raindrops hit the plate, it will start to oscillate and the distance to the coil will change. This change has an influence on the mutual inductance and, consequently, on the resonant frequency of the oscillating circuit.

In addition, the sensor should require little energy which is provided by energy harvesting, and data should be forwarded to a server using wireless data transmission. This allows the sensor to be installed independently of a power connection and a connection to a wired data network.

### 2.2. Calculating the Magnetic Vector Potential and Mutual Inductance

**Figure 2:**
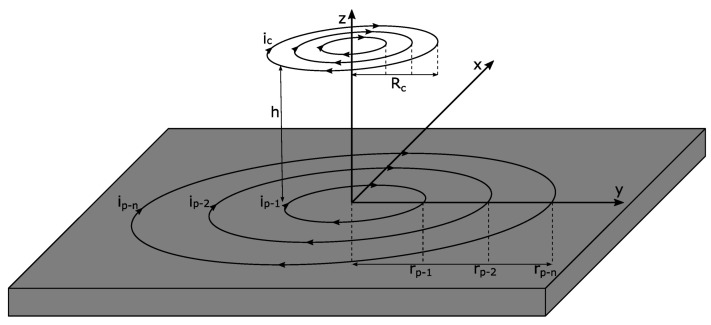
Circular current lines on the plate.

First, the behavior of the coil and the interaction with the metal plate are investigated. In this application the magnetic field is caused by a flat coil and the eddy currents are induced in a metal plate, with both the coil and the plate lying in the x-y plane (Figure 2). The eddy currents and mutual induction highly depend on the distance (h) between the coil and the plate. Table 1 contains a list of the variables used in this work.

The magnetic vector potential caused by primary winding with a current $i_{c}\left(t\right)$ at a point on the plate can be described as follows [21].

(1) $$A_{p}\left(t\right)=\frac{\mu }{4\pi }\int^{\;}\frac{i_{c}\left(t\right)}{\left|R_{c}-r_{p}\right|}dl_{c}$$

If we now consider one coil turn and separate it into radial and azimuth direction, we obtain.

(2) $$A_{r}=-\frac{\mu \cdot i_{c}\left(t\right)}{4\pi }\int_{0}^{2\pi }\frac{R_{c}\cdot \sin\left(\varphi \right)}{\sqrt{R_{c}^{2}+r_{p}^{2}-2\cdot R_{c}\cdot r_{p}\cdot \cos\left(\varphi \right)+h^{2}}}d\varphi$$

(3) $$A_{\sigma }=\frac{\mu \cdot i_{c}\left(t\right)}{4\pi }\int_{0}^{2\pi }\frac{R_{c}\cdot \cos\left(\varphi \right)}{\sqrt{R_{c}^{2}+r_{p}^{2}-2\cdot R_{c}\cdot r_{p}\cdot \cos\left(\varphi \right)+h^{2}}}d\varphi$$

Here, we can already see that the radial component must be zero because it is an antisymmetric function [22]. Introducing $k_{c,p}$ and θ and inserting it into Formula (3), we obtain an elliptic integral whose solution is known [23,24].

(4) $$k_{c,p}=\sqrt{\frac{4\cdot \frac{r_{p}}{R_{c}}}{{\left(1+\frac{r_{p}}{R_{c}}\right)}^{2}+{\left(\frac{h}{R_{c}}\right)}^{2}}}$$

(5) $$\theta =\frac{\varphi -\pi }{2}$$

(6) $$A_{\sigma }=\frac{\mu \cdot i_{c}\left(t\right)}{\pi \cdot k_{c,p}}\cdot \sqrt{\frac{R_{c}}{r_{p}}}\;\cdot [\left(1-\frac{k_{c,p}^{2}}{2}\right)\;\cdot \int_{0}^{\frac{\pi }{2}}\frac{d\theta }{\sqrt{1-k_{c,p\cdot }^{2}\cdot \sin^{2}\left(\theta \right)}}-\int_{0}^{\frac{\pi }{2}}\sqrt{1-k_{c,p\cdot }^{2}\cdot \sin^{2}\left(\theta \right)}d\theta ]$$

Since there is no radial component, it can be assumed that the current on the plate flows only on circular paths. These paths can be considered as individual ring conductors isolated from each other [24]. This results in a transformer arrangement. The excitation coil is the primary side and the rings on the plate are the secondary side. It should be noted that the rings on the secondary side are almost short-circuited.

**Figure 3:**
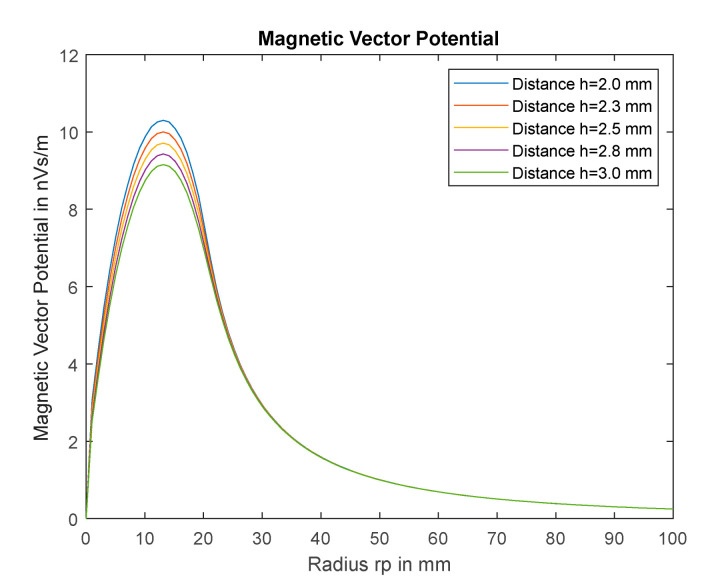
Magnetic Vector Potential for different distances.

To determine the area of effect, the magnetic vector potential on the plate has been calculated. A flat coil with an inner radius of 2 mm, an outer radius of 20 mm and 18 turns with a wire thickness of 1 mm is used as the excitation coil. The specified current is 1 mA. The metal plate is stainless steel type 1.4301 with a permeability of ${µ}_{r}<1.3$ [25]. For simplicity, ${µ}_{r}=1$ is assumed. In Figure 3 it can be seen that the magnetic vector potential reaches its maximum at a radius of 12 mm. Beginning with a radius of 50 mm, the vector potential is only less than 10% of the maximum value and, thus, has hardly any influence on the mutual inductance.

The magnetic flux Φ flowing through the secondary coil creates a mutual inductance M that counteracts the primary inductance [26].

(7) $$\Phi =\oint^{\;}\vec{A}\left(I\right)dl$$

(8) $$M=\frac{\Phi }{I}$$

Inserting (6) into (7) and (7) into (8), we obtain the mutual inductance for each combination of turn and ring. The resulting total mutual inductance can be determined by adding the individual values.

(9) $$M_{ges}=\overset{c}{\underset{1}{\sum }}\overset{p}{\underset{1}{\sum }}\frac{1}{I}\oint^{\;}{\vec{A}}_{c,p}\left(I\right)dl$$

**Figure 4:**
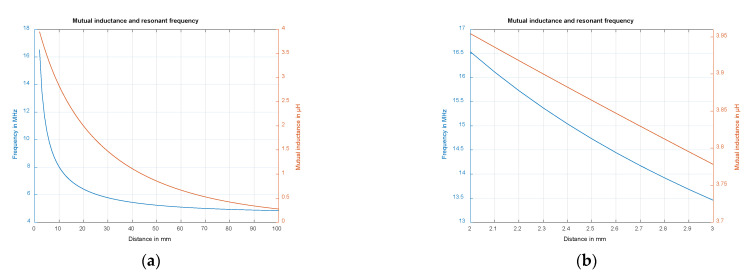
(**a**) Mutual inductance and resonant frequency for distances *h* between 2–100 mm; (**b**) Mutual inductance and resonant frequency for distances *h* between 2–3 mm.

With the calculated mutual inductance, the frequency of the resonant circuit can be expressed as a function of the distance *h*. For this purpose, the inductance and capacitance of the resonant circuit without plate were measured (L=4.3 μH, C=260 nF). Figure 4a shows the resulting mutual inductance and frequency of the resonant circuit over the distance *h* of 2–100 mm and Figure 4b shows the according change in mutual inductance and frequency over 2–3 mm. Therefore, we can assume a nearly linear dependency for small distance changes.

### 2.3. Mechanical Behavior of the Plate

The plate is made of stainless steel 1.4301 and has a thickness of 0.2 mm and a radius of 200 mm. The measurement effect occurs when a raindrop hits the plate, causing it to oscillate and change distance to the coil. The side surfaces of the plate are fully clamped and fixed. Finite element simulations were used to determine the deflection of the plate for a momentum occurring at different points. For this purpose, the momentum caused by a raindrop must first be determined. The momentum depends on the mass and the velocity of the drop. These parameters vary depending on the type of precipitation. Assuming that a raindrop has a spherical volume, the mass can be approximated using the diameter and density of water [27].

(10) $$\vec{p}=m_{d}\cdot \vec{v_{d}}$$

(11) $$m_{d}=\frac{4}{3}\cdot \pi \cdot r^{3}\cdot \rho$$

Light rain with droplet diameters from 0.5 to 1 mm falls at velocities of 3 m/s. A medium raindrop size is in the range of 1–4 mm in diameter and a falling speed of 7 m/s. However, during heavy rain, the drops can reach a diameter of up to 8 mm and a velocity of 13 m/s [28,29]. The momentum for light (0.75 mm diameter), medium (3 mm diameter) and heavy rain was investigated in simulations.

**Figure 5:**
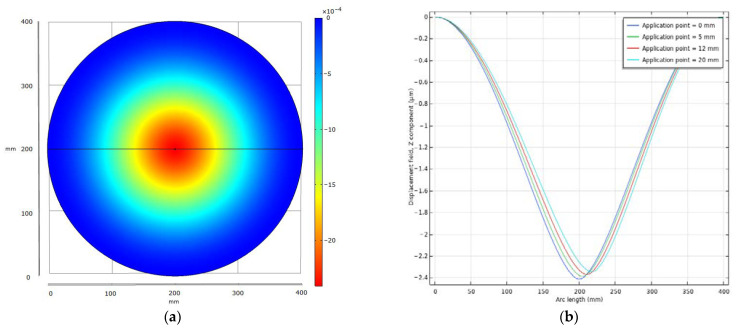
Deflection for light rain. (**a**) Top view of the plate with deflection as a colormap; (**b**) Deflection for different application points along the cross section of the plate.

Figure 5 shows the results for light rain. In (a) the top view of the plate with the deflection is displayed. The point of application is in the center. The course of deflection along the cross-section of the plate for different points of application is shown in (b).

Table 2 shows the deflection at the center and in a distance of 12 mm from the center of the plate for a momentum arising at different points and in different strengths. As previously determined from the magnetic vector potential, the largest influence on mutual inductance due to a change in distance is expected at 12 mm from the center.

Based on the previous calculations for frequency change as a function of distance *h*, an approximation can be made regarding how frequency changes are caused by an impacting raindrop. Assuming that we are in the linear range for small distance changes as shown above, light rain triggers a frequency change of about 2 kHz, medium rain of 15 kHz, and heavy rain of 100 kHz. These approximate values are affected by errors because, as the simulation shows, a drop does not deflect the plate uniformly and also the basic mechanical stresses in the material are not considered. However, they provide a classification of the expected measurement effect and resolution.

## 3. Results and Discussion

### 3.1. Low Power Electronical Measuring Circuit

**Figure 6:**
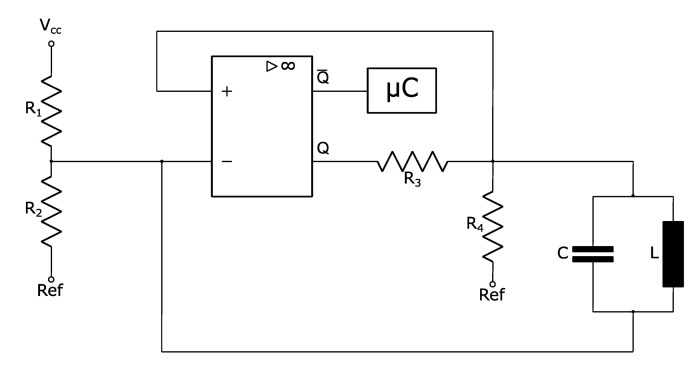
Parallel LC-Circuit with a comparator.

The measuring effect is based on the evaluation of an oscillating circuit frequency. To achieve this, a high-speed and low-power comparator is implemented. The comparator is connected to the excitation coil as shown in Figure 6. To trim the resonant frequency of the oscillating circuit and to reduce interference, a capacitor is connected in addition to the parasitic capacitance of the coil. To lower the current consumption, a parallel LC-oscillator was chosen as it has a high impedance at the resonant frequency. Besides the permanent excitation of the oscillating circuit by the output Q of the comparator, the comparator has an additional output $\bar{Q}$, which is directly connected to a microcontroller. The comparator output $\bar{Q}$ provides a square wave signal, which is already a digital signal. This has the advantage that no AD conversion has to be performed. By counting the edges of the signal, the microcontroller evaluates the frequency. In order to be able to capture “every drop”, the sample time of a measurement is 10 ms with a resolution of 100 Hz and in a measuring range of 200 Hz–30 MHz.

### 3.2. Data Transmission and Power Supply

The data are transmitted via a long-range wide area network (LoRaWAN). With low transmission power and, thus, low power consumption, transmission ranges of up to 10 km can be reached in open areas [30,31]. In the application envisaged here in a densely built-up urban area, ranges of 2–5 km are still possible, so that only a few gateways are required for reception [32]. The frequency is 863–870 MHz with a bandwidth of up to 11 kbit/s [33]. During a rain event, sensor data are sent once per minute. Within dry periods, a status is sent once a day, which also contains diagnostic data such as the current battery level. This additionally reduces maintenance. The data are received via a gateway and then forwarded to a server. The open-source network TTN (The Things Network) was used for the initial tests [34]. The hardware used is the LoRa chip SX1276. The chip supports the European and American radio spectrums for LoRa applications [35].

Attention was paid to a power-saving operation of the system. The electrical circuit without microcontroller requires a current of less than 2 mA. Together with the microcontroller, a current of 30 mA is required. During the sending of data, current peaks of 140 mA occur for a short time. When it is not raining, the microcontroller is put into a deep sleep and the oscillating circuit is switched off. During the deep sleep, a current of less than 1 mA is needed.

The sensor is powered by a lithium-ion battery and a solar module. The power management circuit enables charging currents of up to 100 mA. The energy-autonomous power supply allows flexible installation on roofs, bus stops, lanterns or similar.

### 3.3. Sensor Design

**Figure 7:**
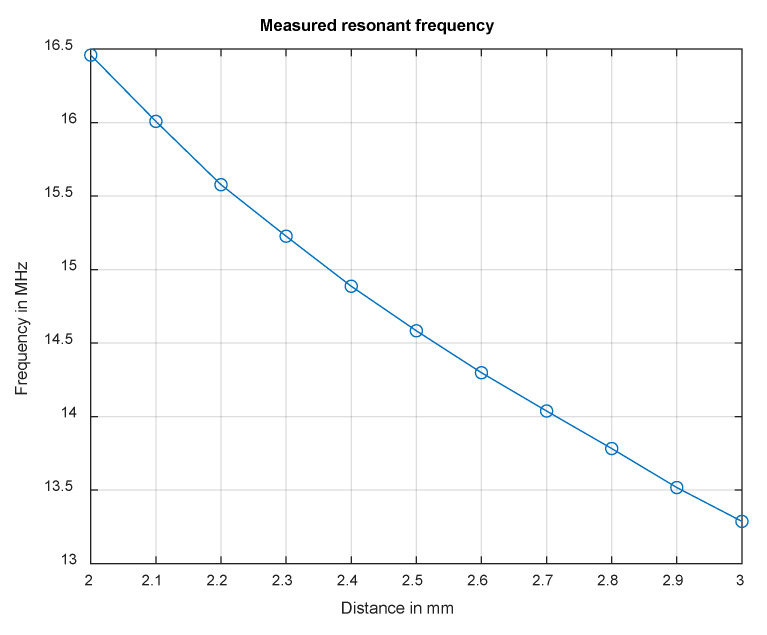
Measured frequency of the resonant circuit for distances between 2–3 mm.

For achieving the optimum distance between the metal plate and the coil, measurements were made. These measurements, which are shown in Figure 7, show the resonant frequency in dependence of the distance between the coil and plate. It becomes obvious that the resonant frequencies are in the same range as the previously calculated values (Figure 4). To determine the optimum distance, the penetration depth of the electromagnetic field must be taken into account. The penetration depth should be less than the thickness of the plate to minimize interference and external influences. The following approximation formula for the skin effect is used for this purpose, with δ being the penetration depth and χ the specific conductivity [36].

(12) $$\delta \;=\sqrt{\frac{2}{\omega {µ}_{r}{µ}_{0}\chi }\;}$$

At more than 14 MHz, the penetration depth is less than half of the thickness of the plate. This shows that we can meet this criterion. However, it is advantageous if the resonant frequency is as high as possible to increase the resolution. For geometrical and design reasons, a minimum spacing of *h* = 2.3 mm must be maintained, resulting in a resonant frequency of about 15.3 MHz.

**Figure 8:**
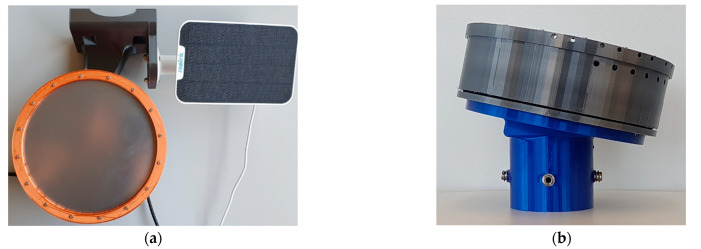
(**a**) Top view of the sensor with solar module; (**b**) Side view of the sensor with 25° inclined mounting base.

The mechanical structure of the sensor is presented in Figure 8. The housing is made of a weatherproof 3D printing material. It also shows the different mounting options. In (a), the mounting bracket is shown, which can be used to attach the sensor to the side of a pole or similar. In (b), the mounting option for positioning the sensor on top of a pole is illustrated. The inclination angle of the sensor of 25°, which is given in both cases, is of importance here. This ensures that the incoming water can run off the plate. Otherwise, the water would accumulate on the plate and emerging raindrops could not be detected clearly.

### 3.4. Single Drop Evaluation

In the laboratory, we were able to produce consistent droplets with a weight of 0.05 g, corresponding to a droplet diameter of about 4 mm. These were dropped onto the sensor from different heights to generate different momentum. In Table 3, the height, velocity and resulting frequency changes are shown.

**Figure 9:**
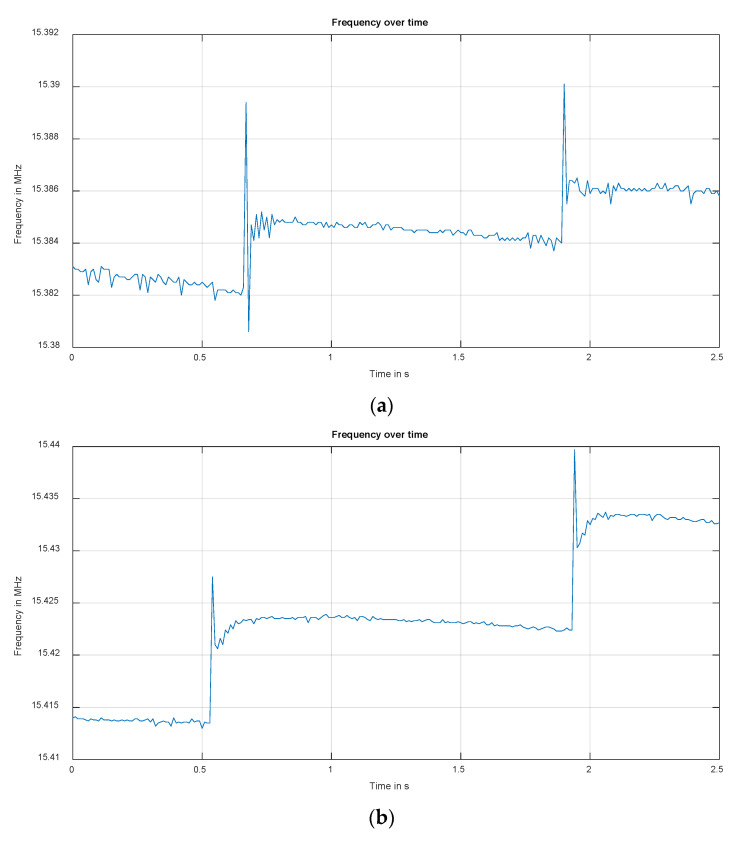

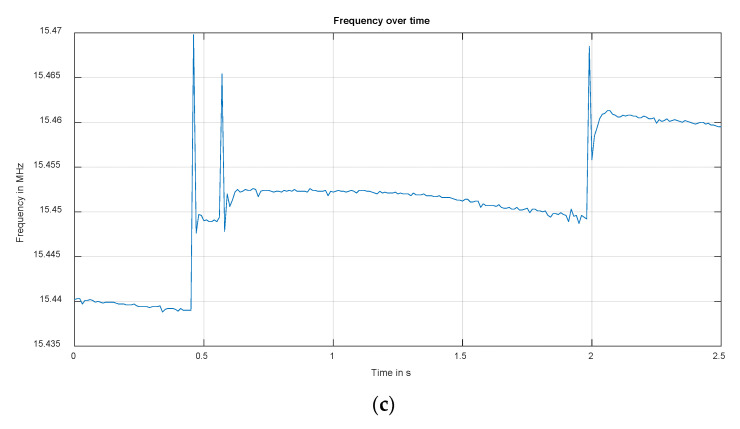
Frequency over time of measurements 1–3.

The unfiltered frequency data are presented in Figure 9. In all three cases, the impacts of the water drops are clearly visible. In (a), the droplet with the lowest velocity, and therefore the lowest momentum, creates a frequency change of about 8 kHz. In (b), a change of 15 kHz and in (c) of 30 kHz can be observed. In addition to the pulse, it can also be seen that the base frequency increases slightly after each drop. This is due to the weight of the drop which does not immediately run off the surface. However, it can be seen that between two drops the base frequency slightly decreases. This is due to the drop moving to the edge of the plate and running off.

## 4. Conclusions

The sensor presented in the results fulfills the requirements formulated in advance. It is possible to operate the sensor self-sufficiently and to install it flexibly. The data are transmitted wirelessly via a LoRaWAN interface. The costs for the sensor have also been kept low. The first demonstrator has a material price of about 150 EUR.

The mathematical considerations carried out in advance are confirmed by the measurements of the frequency as a function of distance. Moreover, the measurements of the single drops show a correlation with the mechanical model. These measurements also prove that it is possible to make a statement about the size of the incoming raindrops.

The measurements of the single drops also offer the possibility to discuss future evaluation methods. A peak analysis could be used to determine the number and size of the drops and to calculate a precipitation value in the common form mm/min. Another approach would be to use a neural network. In this case, a period of measurement data would be collected. Then, a trained neural network could evaluate the precipitation in mm/min. To implement these methods, we need to collect measurement data in advance. For this purpose, the inductive sensor is currently installed next to a high-precision commercial sensor. This is the rain[e] from the company Lambrecht meteo GmbH [37], which is also used by the German Weather Service. In addition, a measuring stand will be set up in order to be able to record measurement data in laboratory as well. These data can then also be used as a reference and to train the neuronal network.

The next steps in this study also include improved shielding of the housing against external fields for reducing interference, since the electromagnetic field of the flat coil cannot propagate to the outside. In addition, the housing should be modified to reduce the angle of inclination required to allow the water to drain. The rain usually does not fall straight down, and if the rain comes from the other side due to wind, detection is limited due to the angle. Furthermore, the angle influences the momentum applied on the plate.

## Figures and Tables

**Table 1:** List of variables and their explanation used in the following.

Variable	Description
A	Magnetic vector potential
$A_{c,p}$	Magnetic vector potential for specific combination of ring on the plate and coil turn
$i_{c}$	Coil current
$R_{c}$	Radius of coil turn
$r_{p}$	Radius of ring on the plate
h	Distance between coil and plate
$\mu =\mu_{0}\cdot \mu_{r}$	Magnetic permeability
Φ	Magnetic flux
M	Mutual inductance
p	Momentum of a raindrop
$m_{d}$	Mass of a raindrop
$v_{d}$	Velocity of a raindrop
ρ	Density of water
δ	Penetration depth
χ	Specific conductivity

**Table 2:** Calculated deflection of the plate for various momentum at varying points of application at different radii.

Precipitation Type	Application Point in Relation to the Center in mm	Deflection in the Center in µm	Deflection at 12 mm from the Center in µm
Light rain	0	2.41	2.35
	5	2.38	2.36
	12	2.33	2.37
	20	2.26	2.33
Medium rain	0	13.71	13.38
	5	13.51	13.42
	12	13.24	13.46
	20	12.82	13.26
Heavy rain	0	98.71	96.35
	5	97.29	96.59
	12	95.34	96.93
	20	92.35	95.47

**Table 3:** Measurement overview.

Measurement	Height in m	Velocity in m/s	Frequency Change in kHz
1	0.1	2.4	~8
2	1.8	5.9	~15
3	2.5	7.0	~30
